# Quantitative CT-Derived Volumetric Bone Mineral Density Threshold for Predicting Cage Subsidence After Oblique Lumbar Interbody Fusion

**DOI:** 10.3390/tomography12050072

**Published:** 2026-05-14

**Authors:** Ji-Le Jiang, Teng-Hui Ge, Zhong-Ning Xu, Jing-Ye Wu, Yu-Qing Sun

**Affiliations:** Department of Spine Surgery, Beijing Jishuitan Hospital, Capital Medical University, No. 31, Xinjiekou East Street, Xicheng District, Beijing 100035, China

**Keywords:** oblique lumbar interbody fusion, cage subsidence, bone mineral density, osteoporosis, intraoperative endplate injury

## Abstract

Background: Cage subsidence (CS) is among the main complications after oblique lumbar interbody fusion (OLIF) and may lead to the failure of indirect decompression. Accurate preoperative bone quality assessment is critical for risk stratification, yet the optimal imaging modality and diagnostic threshold remain unclear. Objectives: This study aimed to determine a quantitative computed tomography (QCT)-derived volumetric bone mineral density (vBMD) threshold for predicting CS after OLIF with posterior fixation. Methods: Patients undergoing OLIF with posterior fixation between July 2017 and March 2020 were retrospectively enrolled. Preoperative vBMD was measured using QCT as the average L2–L4 trabecular volumetric BMD. CS was defined as a loss of more than 2 mm of disk height on sagittal midline CT views between 3 days postoperatively and the last follow-up. Clinical and radiographic parameters including gender, age, body mass index, vBMD, number of operative levels, cage dimensions, disk height, segmental lordosis, intraoperative endplate injury, and fusion status were analyzed. Results: 86 patients (107 operative levels) with a mean follow-up of 20.6 months were included; 25 levels (23.4%) developed CS. Multivariate logistic regression identified vBMD (*p* < 0.001; OR 0.947; 95% CI 0.923–0.972) and intraoperative endplate injury (*p* = 0.031; OR 3.640; 95% CI 1.125–11.776) as independent risk factors. The area under the receiver operating characteristic curve (AUC) for vBMD was 0.847 (95% CI, 0.762–0.932), with an optimal threshold of 83.0 mg/cm^3^ (sensitivity 84.0%, specificity 76.8%). This threshold closely aligns with the American College of Radiology QCT criterion for osteoporosis (80 mg/cm^3^); however, given that it was derived from a single-center retrospective cohort, external validation in multi-center studies is warranted before broad clinical adoption. Fusion rates differed significantly between CS and non-CS groups (84.0% vs. 96.3%, *p* = 0.029). Conclusions: QCT-derived vBMD provides a phantom-calibrated, protocol-standardized metric for preoperative risk stratification of cage subsidence after OLIF.

## Simple Summary

Cage subsidence is a common complication after oblique lumbar interbody fusion surgery, potentially causing loss of spinal alignment and nerve compression. Bone quality is a key risk factor, yet the best way to measure it before surgery remains unclear. We used quantitative CT with a calibration phantom to measure true three-dimensional bone density in 86 patients and found that a bone density below 83.0 mg/cm^3^, combined with surgical damage to the vertebral endplate, strongly predicted cage sinking. This threshold closely matches the established osteoporosis criterion and may serve as a practical, standardized tool for preoperative risk assessment pending multi-center validation.

## 1. Introduction

Oblique lumbar interbody fusion (OLIF), first described by Silvestre et al. [1], has gained widespread acceptance for treating degenerative lumbar diseases owing to its ability to achieve indirect neural decompression and restore segmental alignment through a retroperitoneal pre-psoas approach [1,2]. Despite favorable clinical outcomes, cage subsidence (CS) remains a prevalent complication, with reported incidence rates ranging from 5.5% to 46.7% [3,4,5,6]. CS may result in the loss of restored disk height and segmental lordosis, potentially leading to recurrent neural compression and revision surgery [3,4,5].

The etiology of CS is multifactorial, involving patient-related factors such as age [7,8], bone mineral density (BMD) [4,9], and intraoperative variables including endplate injury [10,11] and cage dimensions [7,10]. Among these, reduced bone quality has been consistently identified as one of the most critical determinants. A recent meta-analysis confirmed that osteoporosis and endplate injury are among the most robust risk factors for CS after OLIF [11]. However, the accuracy and reliability of bone quality assessment largely depend on the imaging modality employed.

Dual-energy X-ray absorptiometry (DXA) remains the current clinical gold standard for osteoporosis diagnosis. However, DXA measures areal BMD (g/cm^2^), which is inherently limited by its two-dimensional projection nature and susceptibility to artifacts from spinal degeneration, osteophytes, aortic calcification, and vertebral compression fractures—conditions highly prevalent in the elderly population undergoing lumbar fusion surgery [12,13,14,15]. Multiple studies have demonstrated that DXA-derived T-scores exhibit only a weak-to-moderate correlation with cage subsidence following lumbar interbody fusion [15,16].

To overcome these limitations, several alternative imaging-based bone quality assessment methods have been proposed. Conventional CT-derived Hounsfield unit (HU) values have emerged as a convenient surrogate marker for BMD, offering the advantage of utilizing preexisting diagnostic CT scans without additional radiation exposure [17,18]. However, HU values are inherently dependent on CT scanner type, tube voltage (kVp), reconstruction kernel, and patient body habitus, resulting in substantial inter-scanner variability that limits standardization across institutions [14,19]. Published HU cutoff values for predicting CS after OLIF vary considerably—ranging from 110 to 155—reflecting this lack of generalizability [17,18,20]. More recently, MRI-based vertebral bone quality (VBQ) scores have also shown promise as radiation-free predictors of cage subsidence [21,22]. Nevertheless, VBQ scores are influenced by MRI acquisition parameters (field strength, sequence type, coil selection) and lack established diagnostic thresholds aligned with internationally recognized osteoporosis criteria [14,23].

In contrast, quantitative computed tomography (QCT) provides true three-dimensional volumetric BMD (vBMD) measurements in standardized physical units (mg/cm^3^), calibrated against a hydroxyapatite reference phantom [24,25]. This calibration renders QCT-derived vBMD largely independent of scanner characteristics, enabling direct comparison across different CT platforms and institutions. Moreover, QCT selectively measures trabecular bone—the metabolically active compartment most sensitive to osteoporotic changes—without confounding contributions from cortical bone or degenerative sclerosis [12,13]. Established diagnostic thresholds from the American College of Radiology (ACR) define osteoporosis as vBMD < 80 mg/cm^3^ and osteopenia as 80–120 mg/cm^3^ [25], providing a standardized framework directly applicable to clinical decision-making.

Despite these theoretical advantages, only a limited number of studies have utilized QCT-derived vBMD to assess the risk of cage subsidence in OLIF [26], and a specific vBMD threshold for predicting CS has not been established. The purpose of this study was therefore to (1) identify independent risk factors for CS after OLIF with posterior fixation using QCT-based vBMD assessment, and (2) determine the optimal QCT-derived vBMD threshold for predicting CS, with the aim of providing a standardized, clinically actionable bone quality metric for preoperative risk stratification.

## 2. Materials and Methods

### 2.1. Patient Population

The present study was a retrospective study including patients who underwent OLIF from July 2017 to March 2020 at a single academic medical institution by one senior spine surgeon. The study was approved by the hospital’s Human Ethics Committee (approval number: 202101-17, approval date: 5 January 2021). The inclusion criteria were as follows: (1) patients who underwent one- or two-level OLIF with bilateral pedicle screw fixation; (2) patients with a diagnosis of degenerative lumbar disease with vertebral instability, including degenerative disk disease, isthmic spondylolisthesis, spinal stenosis, or degenerative spondylolisthesis; (3) patients unresponsive to standard conservative treatment for at least 3 months; (4) patients with a minimum follow-up of one year; (5) patients who had computed tomography (CT) before surgery, 3 days after surgery, and at last follow-up. The exclusion criteria were patients with abnormal endplates, such as previous fracture, previous lumbar fusion, infections, tumors, Modic changes, or Schmorl’s nodes. Data collection included demographic data, cage-related parameters, and radiographic assessment.

### 2.2. Surgical Procedure

The OLIF procedure has been described previously. The polyetheretherketone (PEEK) cages (Clydesdale Spinal System, Medtronic, Memphis, TN, USA) wereinserted into the disk space in each patient. The cages were 6° in lordotic angle, 18 mm in width, 45, 50, 55, or 60 mm in length, and 10, 12 or 14 mm in height. The OLIF cage was filled with demineralized bone matrix and allogeneic bone graft materials. Bilateral pedicle screw fixation was applied in each patient. Laminar fenestration for direct decompression was performed for some patients.

### 2.3. Clinical Assessment

**Figure 1:**
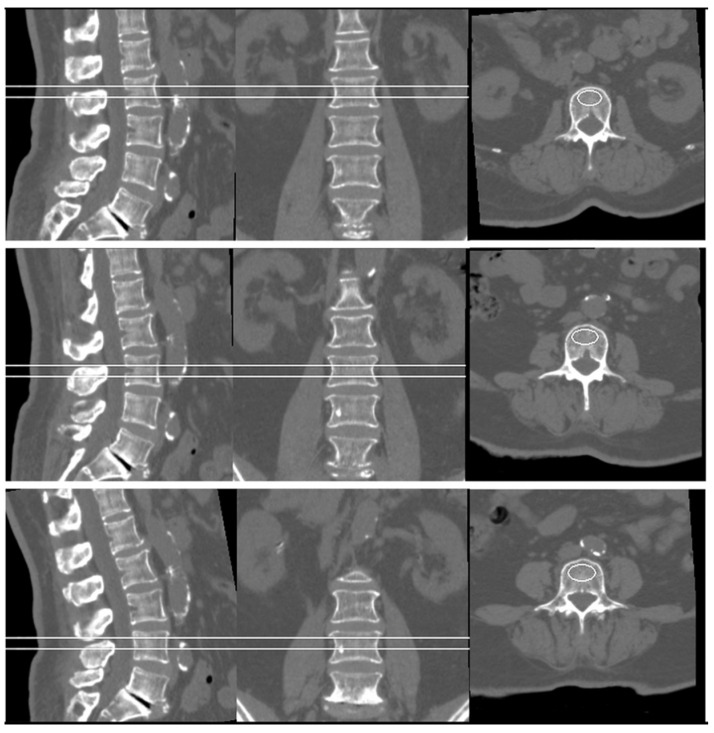
Measurements of the BMD values on preoperative QCT. BMD was calculated as the average among lumbar spine (L2–L4) trabecular volumetric BMD.

Preoperative volumetric BMD was measured using quantitative computed tomography (QCT). CT scans were performed using a Toshiba Aquilion 64-slice CT scanner (Toshiba Medical Systems Corp., Tokyo, Japan) in the supine position. The scanning parameters were as follows: tube voltage, 120 kV; tube current, 250 mA; slice thickness, 1.0 mm; beam pitch, 1.0. A Mindways 5-sample solid-state calibration phantom (Mindways Software Inc., Austin, TX, USA) was placed beneath the patient’s lumbar spine during scanning to enable phantom-based BMD calibration. All CT images were transferred to a dedicated QCT PRO workstation and analyzed using Mindways QCT PRO software (3D Spine function, version 6.1; Mindways Software Inc., Austin, TX, USA). Elliptical regions of interest (ROIs) were automatically placed in the trabecular bone at the midplane of the L2, L3, and L4 vertebral bodies, avoiding cortical bone, the basivertebral venous plexus, and focal sclerotic lesions. The average trabecular vBMD across L2–L4 was calculated and expressed in milligrams per cubic centimeter (mg/cm^3^) of calcium hydroxyapatite equivalent (Figure 1). All QCT measurements were performed by two experienced radiologists who were blinded to clinical outcomes. Osteoporosis was defined as vBMD < 80 mg/cm^3^ and osteopenia as 80–120 mg/cm^3^, according to the American College of Radiology (ACR) criteria [25].

### 2.4. Radiographic Assessment

The radiographic data were independently measured and assessed by two observers who received training on measurement. The average value of two measurements was used for data analysis. The CTs were obtained before surgery, 3 days after surgery, and at the last follow-up. Disk height (DH) and segmental lordosis (SL) were obtained by the midline sagittal CT views. Cage position and intraoperative endplate injury were assessed by the midline sagittal CT views 3 days after surgery. CS and fusion status were assessed by the midline sagittal CT views at the last follow-up. DH was defined as the average of the anterior and posterior heights of intervertebral space. SL was defined as the Cobbs angle between the superior endplate and inferior endplate in the intervertebral space. Cage position is measured as the ratio of the distance between the center of the cage and the anterior edge of the upper endplate to the length of the upper endplate. Intraoperative endplate injury was defined as cage entering into the adjacent cortical endplate more than 2 mm by the midline sagittal CT views 3 days after surgery [27]. Radiographic CS was defined as a loss of more than 2 mm of disk height in sagittal midline computed tomography views between 3 days after surgery and at the last follow-up [28]. Fusion was defined as the bridging bone across the interbody cage. Disk height gap was defined as the difference between cage height and preoperative disk height.

To evaluate inter-observer reliability, all radiographic measurements (disk height, segmental lordosis, cage position, and cage subsidence) were independently performed by two trained observers who were blinded to clinical outcomes. Inter-observer agreement was assessed using the intraclass correlation coefficient (ICC) with a two-way mixed-effects model for absolute agreement and 95% confidence intervals. The average of the two observers’ measurements was used for subsequent statistical analyses.

### 2.5. Statistical Analysis

All statistical analyses were performed with SPSS 26.0. An independent-sample *t*-test and a Chi-squared test were applied to compare the baseline patient characteristics between the CS group and No-CS group. The variables with a *p*-value less than 0.05 in univariate analysis were included in the multivariate logistic regression analysis to identify the independent risk factors for CS. Receiver operating characteristic (ROC) curves were performed to ascertain the most appropriate threshold (cutoff value) of BMD for cage subsidence. A Chi-squared test was used to identify the difference in CS rates among groups. Statistical significance was accepted at *p* < 0.05.

## 3. Results

In this study, 86 patients with 107 OLIF levels were enrolled and 25 levels developed CS, accounting for 23.4% of the total. The mean follow-up time was 20.6 ± 8.0 months. Patient demographics are shown in Table 1. At last follow-up, Fusion rates were significantly different in the two groups (84.0% in the CS group; 96.3% in the No-CS group, *p* = 0.029) and no revision surgery was observed in both groups.

Inter-observer Reliability

Inter-observer agreement for radiographic measurements was excellent. The ICC values were as follows: disk height (ICC = 0.92, 95% CI: 0.91–0.95), segmental lordosis (ICC = 0.93, 95% CI: 0.89–0.95), cage position (ICC = 0.90, 95% CI: 0.85–0.94), and cage subsidence assessment (ICC = 0.92, 95% CI: 0.88–0.93). These results indicate high reproducibility of the radiographic measurements between the two observers.

### 3.1. Univariate Analysis

Univariate analysis was performed for all variables (Table 2). Univariate analysis (Table 2) showed significant differences in BMD (*p* < 0.001) and intraoperative endplate injury (*p* = 0.009).

### 3.2. Multivariate Analysis

In the multivariable analysis, a logistic regression analysis identified BMD (*p* < 0.001; OR 0.947; 95% CI 0.923–0.972) and intraoperative endplate injury (*p* = 0.031; OR 3.640 vs. no injury; 95% CI 1.125–11.776) as independent risk factors (Table 3).

### 3.3. BMD Value, Endplate Injury and Cage Subsidence

**Figure 2:**
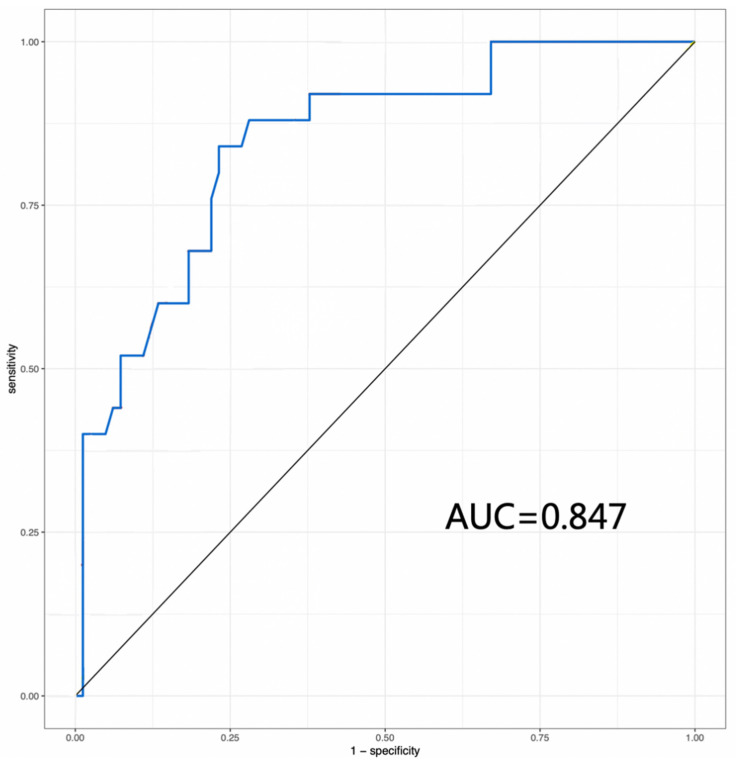
An ROC curve evaluating BMD values and cage subsidence.

**Figure 3:**
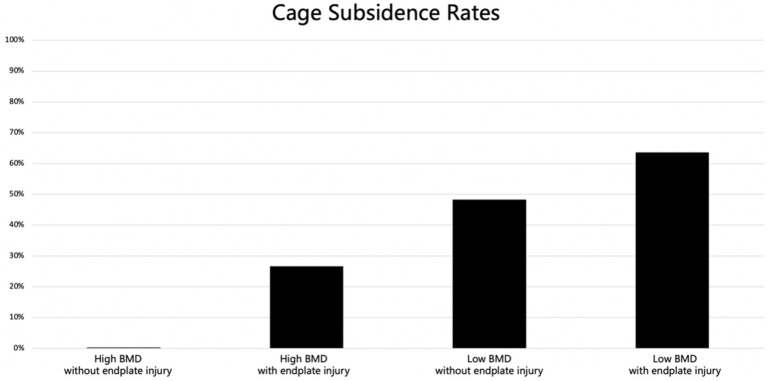
Cage subsidence rates. High BMD was defined as BMD ≥ 83.0 mg/cm^3^; low BMD was defined as BMD < 83.0 mg/cm^3^.

**Figure 4:**
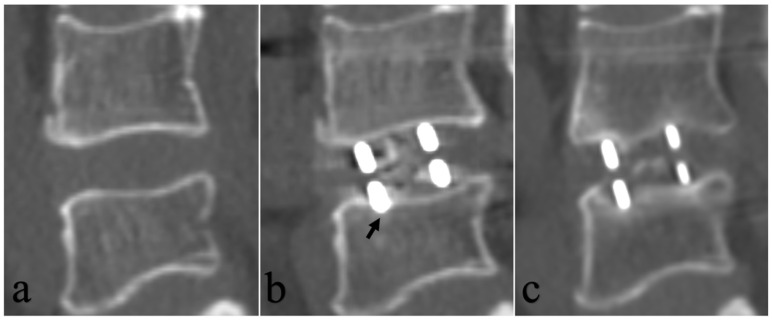
Case 1. Images obtained in a 56-year-old man with BMD (95.0 mg/cm^3^) and presence of intraoperative endplate injury following OLIF with bilateral posterior pedicle screw fixation. (**a**) Preoperative CT sagittal reconstruction. (**b**) CT sagittal reconstruction at 3 days postoperatively showing intraoperative endplate injury (the area of endplate injury indicated by arrow). (**c**) CT sagittal reconstruction at 3.3 years postoperatively showed the presence of CS.

**Figure 5:**
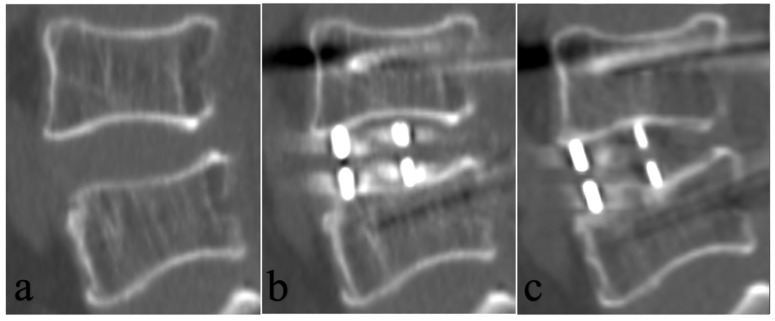
Case 2. Images obtained in a 63-year-old woman with BMD (41.3 mg/cm^3^) and absence of intraoperative endplate injury following OLIF with bilateral posterior pedicle screws fixation. (**a**) Preoperative CT sagittal reconstruction. (**b**) CT sagittal reconstruction at 3 days postoperatively. (**c**) CT sagittal reconstruction at 2.5 years postoperatively showed the presence of CS.

An ROC curve of BMD value was performed to develop separation criteria between CS and No-CS, with an area under the curve of 0.847 (95% CI: 0.762−0.932) (Figure 2). The most appropriate threshold of the BMD value to predict the incidence of CS was 83.0 mg/cm^3^ (sensitivity 84.0%, specificity 76.8%), which defined high BMD and low BMD. There was a statistically significant difference in CS rates among the four groups based on BMD and endplate injury (*p* < 0.001) (Table 4, Figure 3). Two representative case examples are presented (Figure 4 and Figure 5).

## 4. Discussion

In this study, we demonstrated that QCT-derived volumetric BMD and intraoperative endplate injury are independent risk factors for cage subsidence after OLIF with posterior fixation, and established a vBMD threshold of 83.0 mg/cm^3^ with good predictive performance (AUC = 0.847, sensitivity 84.0%, specificity 76.8%). To our knowledge, this is among the first studies proposing a specific QCT-derived vBMD cutoff value for predicting CS in OLIF patients with supplemental posterior fixation.

### 4.1. Incidence of Cage Subsidence

The CS incidence in our cohort was 23.4% (25/107 levels), consistent with previously reported rates of 5.5–46.7% for lateral lumbar interbody fusion with supplemental posterior fixation [6,26,28,29,30]. A recent systematic review and meta-analysis by Shen et al. [11] confirmed that CS remains a common complication even with posterior instrumentation, underscoring the need for reliable preoperative risk stratification tools. It is important to distinguish between radiographic (mechanical) cage subsidence and clinically relevant subsidence. In our cohort, the significantly lower fusion rate in the CS group (84.0% vs. 96.3%, *p* = 0.029) provides indirect biomechanical evidence that subsidence at this threshold has biological consequences beyond pure imaging artifact. However, the absence of patient-reported outcome measures (PROMs) such as the Visual Analog Scale (VAS), Oswestry Disability Index (ODI), and SRS-22 in this dataset precludes formal stratification into asymptomatic versus symptomatic subsidence. We acknowledge that the relationship between mild radiographic subsidence and patient-reported outcomes in the published OLIF literature remains inconsistent, and that the present study cannot directly resolve this question.

Our study identified vBMD as the strongest independent predictor of CS. This finding aligns with the well-established relationship between low bone quality and cage subsidence [4,9,11,31]. However, the imaging modality used to assess bone quality critically determines the accuracy and clinical utility of the measurement.

DXA, despite being the clinical gold standard for osteoporosis diagnosis, has well-documented limitations in the degenerative lumbar spine. Areal BMD measurements by DXA are confounded by osteophytes, facet joint hypertrophy, aortic calcification, and endplate sclerosis, frequently leading to overestimation of true bone density and underdiagnosis of osteoporosis [12,13,15]. A recent study comparing HU, VBQ, and DXA T-scores for predicting cage subsidence after posterior lumbar interbody fusion demonstrated that DXA exhibited the lowest predictive accuracy among the three modalities [16]. Similarly, in OLIF-specific research, DXA T-scores showed only modest correlation with the degree of cage subsidence (AUC approximately 0.695–0.791) [15,18].

CT-derived HU values have gained popularity as an opportunistic bone quality metric, leveraging preexisting diagnostic CT without additional radiation or cost [17,18,20]. Several studies have reported HU cutoff values for predicting CS after OLIF: Ran et al. [17] identified a threshold of HU < 135 (AUC = 0.82), Pu et al. [18] reported an L1-L4 horizontal HU threshold with AUC of 0.909, and a recent study [20] utilized CT attenuation value classification to stratify subsidence risk. However, a systematic review and meta-analysis by [23] encompassing 28 studies and 3729 patients demonstrated that HU values, while moderately predictive (pooled AUC approximately 0.812), suffer from significant inter-study heterogeneity attributable to differences in CT scanners, tube voltage settings, reconstruction algorithms, and measurement protocols. This scanner-dependency fundamentally limits the generalizability of HU-based cutoff values across institutions.

MRI-based VBQ and endplate bone quality (EBQ) scores represent another emerging approach [21,22]. Pu et al. [27] directly compared HU values and VBQ scores for predicting CS after OLIF and found comparable predictive performance. Zheng et al. [21] further evaluated site-specific MRI-based bone quality assessments in OLIF patients. While these MRI-derived scores offer the advantage of avoiding ionizing radiation, they lack standardization against established osteoporosis diagnostic criteria and remain influenced by MRI acquisition parameters.

QCT-derived vBMD addresses these limitations through phantom-calibrated, three-dimensional trabecular bone density quantification in absolute physical units (mg/cm^3^) [12,24,25]. Unlike HU values, QCT-derived vBMD is calibrated against a standardized reference phantom, substantially reducing inter-scanner variability and enabling cross-institutional comparability. Unlike VBQ scores, QCT-vBMD can be directly referenced to established ACR diagnostic thresholds for osteoporosis (<80 mg/cm^3^) and osteopenia (80–120 mg/cm^3^) [26]. Notably, the optimal vBMD threshold identified in our study (83.0 mg/cm^3^) closely aligns with the ACR osteoporosis threshold of 80 mg/cm^3^, providing clinicians with a standardized, externally validated reference point directly integrable into existing diagnostic workflows. This congruence between our data-driven cutoff and the established ACR criterion strengthens the clinical credibility and practical applicability of our findings. These methodological characteristics confer theoretical and complementary advantages over DXA, HU, and VBQ; however, the relative predictive superiority of QCT-vBMD remains to be empirically established through head-to-head intra-cohort comparison.

It is important to acknowledge that the 83.0 mg/cm^3^ threshold derived from our cohort, while statistically optimal for our population, requires external validation before generalization. The close concordance between our data-driven cutoff and the long-established ACR osteoporosis threshold (80 mg/cm^3^) is reassuring and provides indirect cross-cohort convergence; however, this congruence should not be misinterpreted as a substitute for prospective multi-center confirmation.

A recent large-scale study [32] analyzing 337 OLIF segments (674 endplates) also utilized QCT-derived vBMD and confirmed its importance as a key predictor, further developing a Least Absolute Shrinkage and Selection Operator (LASSO)-based nomogram prediction model. While their advanced machine learning approach incorporated multiple endplate morphology variables, our study provides a simpler, clinically actionable threshold (83.0 mg/cm^3^) combined with intraoperative endplate injury assessment, which may be more readily applicable in routine clinical settings where complex prediction algorithms are not available.

### 4.2. Intraoperative Endplate Injury

The second independent risk factor identified in our study was intraoperative endplate injury, consistent with prior biomechanical and clinical evidence [10,11,33]. Santoni et al. [33] demonstrated in a cadaveric study that endplate violation compromises segmental stability following lateral cage placement. Tohmeh et al. [10] reported that operative levels with endplate injury had significantly more severe late-onset CS. Our findings, combined with the CT-based assessment of endplate integrity on postoperative imaging, reinforce the importance of meticulous surgical technique to preserve cortical endplate integrity and the value of early postoperative CT in identifying endplate violations that may predispose to subsequent subsidence.

### 4.3. Clinical Implications of Combined BMD and Endplate Assessment

The stratified analysis based on both vBMD (above vs. below 83.0 mg/cm^3^) and endplate injury status revealed significant differences in CS rates among the four subgroups (Table 4), suggesting that the combination of preoperative QCT-based bone quality assessment with postoperative CT evaluation of endplate integrity provides a practical, imaging-based risk stratification framework. For patients with vBMD < 83.0 mg/cm^3^, preoperative bone health optimization—including initiation of anti-osteoporotic pharmacotherapy and, when feasible, surgical postponement until biochemical and densitometric improvement—should be considered.

### 4.4. Impact on Fusion

Our study demonstrated that fusion rates were significantly lower in the CS group compared with the non-CS group (84.0% vs. 96.3%, *p* = 0.029), aligning with prior studies [24,30]. This finding underscores the clinical relevance of cage subsidence beyond mere radiographic observation, as compromised fusion may contribute to persistent symptoms and potential need for revision surgery [5].

### 4.5. Future Directions

Looking forward, the cross-scanner reproducibility of phantom-calibrated QCT-vBMD positions it favorably for translation into a generalizable clinical guideline. We are currently planning a prospective multi-center study involving four tertiary spine centers using diverse CT platforms (GE, Siemens, Philips, and Toshiba), with a uniform Mindways calibration protocol, to externally validate the 83.0 mg/cm^3^ threshold and to determine whether minor population-specific adjustments (e.g., for ethnicity-related trabecular density variation) are warranted. If successful, such validation could elevate this threshold from a single-institution finding to a broadly applicable preoperative risk-stratification standard for OLIF.

### 4.6. Limitations

This study has several limitations. First, this was a retrospective single-center study, which may introduce selection bias. Second, as only OLIF cages with 6° of lordosis and 18 mm of width were available at our institution, we were unable to assess the effect of cage angle and width on CS. Third, we only selected patients with short-segment OLIF surgery to avoid metal artifacts and obtain accurate measurements on sagittal midline CT views, which precluded evaluation of multi-level OLIF surgery. Fourth, the present study did not perform a head-to-head comparison of QCT-derived vBMD with DXA T-scores, CT-derived HU values, or MRI-based VBQ scores within the same cohort—limitations imposed by the retrospective design and incomplete availability of contemporaneous DXA and standardized MRI sequences across the entire cohort; we recognize this as a substantive limitation. Such a head-to-head comparison is the central aim of our planned follow-up investigation. Fifth, the threshold of 83.0 mg/cm^3^ was derived from a single-center cohort and requires external validation in multi-center studies with diverse CT platforms before widespread clinical adoption. Sixth, patient-reported outcome measures (VAS, ODI, SRS-22) and functional assessments were not systematically captured in this retrospective database, which prevents differentiation between asymptomatic mechanical subsidence and clinically symptomatic subsidence. Finally, formal inter-observer ICC for QCT-derived vBMD was not computed; prospective dual-reader ICC will be reported in our planned validation study. Additionally, preoperative anti-osteoporotic medication use and serum bone-turnover markers were not available in this cohort and should be prospectively collected in future studies.

## 5. Conclusions

In this study, QCT-derived volumetric BMD and intraoperative endplate injury were identified as independent risk factors for cage subsidence after OLIF with posterior fixation. The optimal vBMD threshold for predicting CS was 83.0 mg/cm^3^, closely aligned with the ACR QCT criterion for osteoporosis, supporting its standardized clinical applicability. Notably, the combination of low vBMD and intraoperative endplate injury produced a markedly elevated subsidence rate of 63.6%, more than two-fold higher than that observed with low vBMD alone, underscoring the synergistic contribution of compromised bone quality and surgical technique. Preoperative QCT-based risk stratification combined with meticulous endplate-sparing surgical technique is therefore essential, and external validation of the 83.0 mg/cm^3^ threshold in multi-center cohorts is warranted before its broad clinical application. 

## Figures and Tables

**Table 1:** Patient demographics (*n* = 86 patients).

Characteristic	Patients (*n* = 86)
Age (years)	60.1 ± 9.8
Gender (female/male)	53/33
BMI (kg/m^2^)	25.9 ± 3.2
BMD (mg/cm^3^)	101.6 ± 38.8
Smokers	8 (9.3%)
Diabetes	10 (11.6%)
Follow-up time (months)	20.6 ± 8.0
Diagnosis	
Degenerative disk disease	16 (18.6%)
Spinal stenosis	19 (22.1%)
Degenerative spondylolisthesis	47 (54.7%)
Isthmic spondylolisthesis	4 (4.7%)
Number of operative levels	
Single-level surgery	65 (75.6%)
Double-level surgery	21 (24.4%)

BMI: Body mass index; BMD: bone mineral density.

**Table 2:** Univariate analyses between CS group and No-CS group. (*n* = 107 operative levels).

Variables	CS Group (*n* = 25)	No-CS Group (*n* = 82)	*p*-Value
Clinical parameters			
Age (years)	64.0 ± 8.4	60.0 ± 9.7	0.066
Gender (female/male)	14/11	50/32	0.657
Smokers	1 (4.0%)	8 (9.6%)	0.364
Diabetes	2 (8.0%)	9 (11.0%)	0.668
BMI (kg/m^2^)	25.6 ± 3.0	25.8 ± 3.3	0.716
BMD (mg/cm^3^)	69.1 ± 23.5	111.2 ± 36.3	<0.001 *
Follow-up time (months)	22.6 ± 7.8	20.0 ± 7.7	0.149
Surgical parameters			
Number of operative levels			
Single-level surgery	13 (52.0%)	52 (63.4%)	0.306
Double-level surgery	12 (48.0%)	30 (36.6%)	
Decompression method			
Indirect decompression	11 (44.0%)	48 (58.5%)	0.201
Direct decompression with fenestration	14 (56.0%)	34 (41.5%)	
Radiographic parameters			
Preoperative			
Disk height (mm)	8.7 ± 2.4	8.2 ± 2.6	0.358
Segmental lordosis (°)	8.0 ± 5.7	7.8 ± 4.7	0.843
Spondylolisthesis	12 (48.0%)	41 (50.0%)	0.861
Postoperative			
Disk height (mm)	11.9 ± 1.6	11.8 ± 1.8	0.767
Segmental lordosis (°)	8.7 ± 4.0	9.6 ± 3.8	0.351
Intraoperative endplate injury	11 (44.0%)	15 (18.3%)	0.009 *
Last follow-up			
Disk height (mm)	9.1 ± 1.5	10.7 ± 1.7	<0.001 *
Segmental lordosis (°)	7.3 ± 5.2	9.2 ± 4.0	0.061
Fusion status	21 (84.0%)	79 (96.3%)	0.029 *
Cage-related parameters			
Cage level			
L2–3	1 (4.0%)	1 (1.2%)	0.574
L3–4	6 (24.0%)	16 (19.5%)	
L4–5	18 (72.0%)	65 (79.3%)	
Cage height			
10 mm	1 (4.0%)	10 (12.2%)	0.151
12 mm	17 (68.0%)	61 (74.4%)	
14 mm	7 (28.0%)	11 (13.4%)	
Cage length			
45 mm	2 (8.0%)	4 (48.8%)	0.095
50 mm	9 (36.0%)	34 (41.5%)	
55 mm	9 (36.0%)	40 (48.8%)	
60 mm	5 (20.0%)	4 (4.9%)	
Disk height gap (mm)	3.8 ± 2.3	3.9 ± 2.4	0.818
Cage position (%)	47.0 ± 7.7	47.1 ± 7.8	0.927

BMI: Body mass index; BMD: bone mineral density. * Means statistically significant.

**Table 3:** Multivariate logistic regression analysis to determine risk factor for cage subsidence (*n* = 107 operative levels).

Parameters	Odds Ratio	Lower 95% CI	Upper 95% CI	*p*-Value
BMD (mg/cm^3^)	0.947	0.923	0.972	<0.001 *
Intraoperative endplate injury (ref: absent)	3.640	1.125	11.776	0.031 *

BMD: Bone mineral density. * Means statistically significant.

**Table 4:** Subsidence rates.

	Cage Subsidence	No Cage Subsidence	Sum	Subsidence Rates	*p*-Value
High BMD without endplate injury	0 (0.0%)	52 (63.4%)	52	0%	<0.001 *
High BMD with endplate injury	4 (16.0%)	11 (13.4%)	15	26.7%	
Low BMD without endplate injury	14 (56.0%)	15 (18.3%)	29	48.3%	
Low BMD with endplate injury	7 (28.0%)	4 (4.9%)	11	63.6%	

* Means statistically significant. High BMD was defined as a BMD ≥ 83.0 mg/cm^3^; Low BMD was defined as a BMD < 83.0 mg/cm^3^.

## Data Availability

The data presented in this study are available on request from the corresponding author; the data are not publicly available due to privacy or ethical restrictions.

## Abbreviations

OLIF	oblique lumbar interbody fusion
CS	cage subsidence
BMD	bone mineral density
QCT	quantitative computed tomography
DXA	dual-energy X-ray absorptiometry
BMI	body mass index
DH	disk height
SL	segmental lordosis
